# Economic losses or environmental gains? Framing effects on public support for environmental management

**DOI:** 10.1371/journal.pone.0220320

**Published:** 2019-07-25

**Authors:** Alexander H. DeGolia, Elizabeth H. T. Hiroyasu, Sarah E. Anderson

**Affiliations:** Bren School of Environmental Science and Management, University of California Santa Barbara, Santa Barbara, California, United States of America; University of Waikato, NEW ZEALAND

## Abstract

Environmental managers face major challenges related to project implementation and communicating the significance of those projects to the public. Effective communication can mitigate public opposition or increase support for specific projects and increase public and political support for environmental management more generally. In this study, we evaluate which types of benefits or losses environmental managers should communicate and how to frame those attributes to achieve greater public support. To do so, we field a survey experiment that presents the benefits of an invasive species management project, utilizing a two (economic, ecological) by two (gain, loss) factorial design as well as a control message. Ecological messages lead to significantly more support for invasive species management than economic messages, and loss frames are more effective than gain frames. We also find that treatment responses differ across several covariates including political ideology and environmentalism. These results indicate that the public is more concerned with managing invasive species for intrinsic environmental worth than economic benefit and that preventing further environmental degradation is more motivating than promoting additional environmental gains.

## Introduction

Active management of environmental resources offers substantial and diverse public benefits, including ecosystem services [1–3]. How environmental managers should communicate the worth of their work to maximize public support, however, remains poorly understood. Although the majority of the public supports broad goals related to environmental protection, a sizable minority remains either skeptical of government intervention to manage environmental quality or does not prioritize the issue [4]. Public opinion on environmental policies is important because it can play a meaningful role in their success or failure [4–6]. Policymakers are typically responsive to mass public opinion [7], and in some cases public support or opposition to environmental programs can play an important role in determining policy outcomes [8–11]. Developing better ways for public agencies and their advocates to communicate about management decisions will increase support for such projects and help to ensure future funding and successful implementation.

Ecological and economic benefits represent two primary goals of environmental management. As a result, they are also two of the most common arguments in support of environmental protection; further, the public is generally familiar with the tenets of environmental policy arguments that focus on ecological and economic impacts [12,13]. Ecological message frames highlight the importance of protecting the environment for the sake of its animals and ecosystems (e.g., to protect endangered species). Economic message frames focus on how environmental protection can benefit human economic activity (e.g., to protect agricultural crops). These same messages can be framed as either providing opportunities for environmental gains or protecting against losses (e.g., gaining habitat or losing agricultural crops).

In this paper, we present the results of a survey experiment of California residents (N = 1077) that utilizes a two (economic, ecological) by two (gain, loss) design to evaluate the impacts of each of these message frames on support for invasive species management. The factorial design of the experiment allows us to determine whether gain and loss frames interact with ecological and economic frames as well as how each performs independently. We also evaluated the role of other covariates such as environmental values and political ideology as predictors of support for management. Whereas existing research regarding the efficacy of these frames on public opinion is almost entirely focused on climate change [14,15], this study provides new information for environmental managers and advocates regarding how people respond to messages about important but comparatively low-profile and non-partisan environmental policy issues.

### Using frames in environmental communication

Environmental management suffers when agencies are unable to communicate the importance of their environmental programs in ways that convince the public or their elected representatives of the value of those programs [16,17]. The public also frequently misunderstands management goals, which can lead to confusion and lack of support [18]. One way to overcome misunderstanding and lack of concern for environmental management is through effective framing, which highlights information that connects to people’s core concerns or beliefs.

Frames contextualize policy issues, making them more immediately accessible and more relevant and understandable for the public [19–22]. By altering the context in which policy choices are presented, frames make those choices more relevant and understandable for the public [19–22]. To better understand how frames influence support for environmental management, we evaluate the impacts of two distinct frame types: attribute frames, which highlight specific factors present in the issue being evaluated, and outcome frames, which present attributes in terms of their promised gains or prevented losses [14,23–25].

#### Using message frames to promote invasive species management

This study evaluates the effect of message frames on support for environmental management using the case of invasive species management in California. Invasive species have major ecological and economic impacts; they can have severe consequence on ecosystems, biodiversity, and economies [26–28]. Terrestrial species are especially impacted by invasive species, and over 900 species globally are documented as being directly affected by invasive species presence [29]. Invasive predators are a major driver of global biodiversity loss, and have been implicated in the extinctions of 87 bird, 45 mammal, and 10 reptile species, and endangering 596 other species [30]. Invasive species also influence delivery of ecosystem services that contribute to human economic activity, with estimates that $120 billion would be needed annually to mitigate invasive species impacts worldwide [28]. More recent estimates focused on invasive insects suggest that mitigation, damage, and human health costs are an estimated $77 billion per year globally [26].

In addition to the major economic and ecological consequences of species invasion, the relatively non-partisan nature of the issue to date and its similarities with other environmental issues make it an excellent case for evaluating environmental communications. Like most environmental issues, the majority of the public supports some type of invasive species management [31,32]. Also like many other environmental issues, few people outside of the scientific or professional environmental community are aware of the scope of invasive species problems or how to address them [8,32–34].

While issues like climate change and energy exploration are prominent in current national partisan rhetoric, most environmental issues–including invasive species management–are not [35,36]. Invasive species management is a useful example of how communications influence public opinion because the public does not hold strong opinions regarding invasive species management [31], leaving room for influence. As a result, we believe that evaluation of support for invasive species management provides significant new information regarding how people interpret environmental communications, which influences public support or opposition for such projects [8,18,37–39].

#### Framing management goals: Ecological and economic messages

In this study, attribute frames differ based on the ecological or economic impacts highlighted in communicating the goals of an invasive species management project. Past environmental communications research has evaluated how certain attribute frames influence environmental attitudes, behavior change, and policy support [14,15,22,40–42]. However, little research has evaluated whether message frames that highlight broad benefits to human economic activity are more effective than frames highlighting ecological system impacts in building public support for environmental policies. Despite this, the common way that environmental policies are communicated as part of public discourse focuses on either the ecological or economic impacts of management, often pitting one against the other.

Economic message frames promote environmental management based on economic co-benefits, which can be effective because economic issues are much more immediately concerning to most Americans than environmental issues [43]. Historically, 20 to 80% of Americans cite economic issues as the Most Important Problem (MIP) facing the country, as compared to about 1 to 5% who cite environmental issues as the MIP [4,43]. For all of these reasons, framing environmental issues as economic opportunities can be an effective tool for promoting environmental protection [22]. Economic arguments re-frame the conversation to highlight co-benefits rather than direct ecological benefits of action, a tactic policy advocates have attempted with some success across a number of policy issues, most notable climate change communication [44,45]. However, in some cases focusing on co-benefits of environmental protection can diminish the perceived urgency of environmental issues [46].

Ecological messages promote environmental protection based on its direct benefits to nature. In many cases, protecting nature is clearly the primary and most important reason for environmental management, while attempting to reframe arguments for economic co-benefits can make protecting nature appear less important [47]. Moreover, for environmental issues like invasive species management in which there are neither dominant frames nor obvious partisans on each side of the debate to react to those frames, identifying economic co-benefits of environmental management is likely to be less important. In these cases, messages targeting the most obvious and direct reasons for certain environmental management decisions are likely to be more successful than messages that identify wide-ranging co-benefits, such as economic development. This leads to our first hypothesis:

*H1*: People will be more supportive of invasive species management when framed as providing ecological benefits than providing economic benefits.

Economic and ecological message frames are interpreted differently by people based on their political and environmental values. This difference is at least in part based on different systems of morality. While conservatives tend to believe in use of the environment for human benefit and in market-based systems that evaluate the value of the natural environment for its human use rather than its intrinsic value [36,42,48], the progressive moral system prioritizes empathy and feelings of responsibility toward others, including non-human others [42,49,50]. As a result, liberals are more likely to believe protecting the environment is a moral responsibility [51,52], which also makes them more responsive to messages that highlight ecological benefits rather than economic ones [53,54]. The result is that liberals should be more responsive to frames that highlight the ecological benefits of environmental policies. This leads to two additional hypotheses:

*H2*: Among liberals, ecological frames will increase support for invasive species management more than economic frames.

*H3*: Among conservatives, economic frames will increase support for invasive species management more than ecological frames.

People’s environmental values also play an important role in dictating how they respond to different messages related to invasive species management. The dissonance associated with harming animals to help human economic growth may be particularly strong among environmentalists, and thus the opportunities to minimize that dissonance through promoting ecological benefits will also be greater. This leads to two additional hypotheses:

*H4*: Among environmentalists, ecological frames will increase support for invasive species management more than economic frames.

*H5*: Among non-environmentalists, economic frames will increase support for invasive species management more than ecological frames.

#### Framing management outcomes: Gain and loss messages

This study also evaluates the impacts of different outcome frames, which present benefits of environmental management goals in terms of preventing losses or facilitating gains. For example, removal of an invasive species can be interpreted as providing opportunities for native species to flourish. Here we interpret recovery to a more ecologically balanced state from the current invaded one as a gain frame because it invokes movement toward environmental goals. Alternatively, removal of an invasive species may provide opportunities to avert further loss of native species. This loss frame highlights the ability of environmental managers to prevent further degradation of the natural ecosystem.

Expectations regarding how people will react to gain and loss messages are rooted in prospect theory [14,23–25]. Prospect theory proposes that people are more responsive to potential losses than equivalent potential gains–the psychological effect of losing $100 is greater than the positive effect of gaining $100. The present research diverges from traditional prospect theory in that we evaluate whether people respond to gains and losses of public rather than private goods. Evaluation of how people respond to messages that highlight proposed gains versus prevented losses can provide essential insight regarding how prospect theory applies broadly to environmental public goods problems.

Existing research regarding the effects of outcome frames on environmental attitudes indicate that gain frames are typically more effective than loss frames for increasing positive attitudes toward climate change mitigation efforts, but loss frames tend to be more effective for increasing concern or behavior for other environmental issues [41,55–61]. One reason for this difference may be that loss frames typically work by increasing the salience and perceived consequence of an issue more than commensurate gain frames [61,62]. Our next hypothesis follows from the fact that invasive species management is not a highly salient issue for most Americans:

*H6*: People will be more supportive of invasive species management when presented in terms of preventing losses (either ecological or economic) than in terms of offering comparable gains.

People may respond to gain and loss frames for certain environmental issues or policies differently based on how they perceive risks associated with the new policy. Under the status quo, people know what to expect, while the alternative choice may be more likely to lead to negative consequences even if it also offers possible gains [63]. However, when faced with potential significant losses, people tend to be risk-seeking (e.g., support a political challenger or major policy change) if they believe the potential losses from the status quo are greater than potential losses from the alternative. One proposed reason for the ineffectiveness of loss frames to motivate action on climate change [14] is that inaction in the face of climate change (the status quo position) is perceived as highly uncertain and potentially very negative [64,65]. Enacting policies to mitigate climate change is perceived as the more cautious approach. Thus, gain frames would be most likely to motivate a risk-averse choice, which in the case of climate change is policy action.

Inaction with respect to invasive species appears a priori to be a riskier choice than non-action because the result could be large ecological and economic damages [66–68]. However, in comparison to climate change, the threats posed by invasive species are both smaller in scale and more likely to be misunderstood. As a result, we expect that most people will maintain a conventional perception of new invasive species policy action as the riskier choice, which will make loss frames more effective at eliciting support for invasive species management than gain frames. Based on both perceived risks and salience, we anticipate that people will be more likely to support invasive species management when framed in terms of potential losses avoided.

We do not anticipate significant differences in outcome frame treatment effects among different subgroups. While some evidence suggests conservatives and Republicans are more responsive to gain-framed messages than to messages that focus on the risk of environmental loss due to inaction [48,51,69], we expect this is an artifact of backlash against partisan rhetoric rather than a natural predisposition to respond to gain frames rather than loss frames. Given the non-partisan nature of invasive species management, we do not anticipate political affiliations to substantially influence differential responses to gain and loss frames.

## Data and methods

### Procedure

This research was approved by the institutional review board of the UC Santa Barbara Office of Research (IRB #44-17-0189). Participants did not provide consent as no personal identifying information was collected and data were analyzed anonymously. A sample of California residents (*N* = 1077) were recruited using an online panel provided by Qualtrics fielded from February 22 to March 16, 2017. The sample was gathered using online quota sampling that allowed collection of a sample of California residents that was representative of the state population in terms of both household income and political party affiliation measures. We also used the quota to oversample rural residents as part of a separate analysis that will not be discussed in detail in this paper [70]. The age of participants closely approximated the California population, while the sample overrepresented women, better educated residents, and white residents. A complete data file with survey results can be found in S1 Data, and a review of sample demographic characteristics is provided in S1 Fig.

We performed all analyses using both an unweighted and weighted sample, with the weighted sample including a number of design and post-stratification weights to compensate for our over-sampling of rural residents and the fact that our sample over-represented women and college-educated people as compared to the California population. Analysis showed no significant change in the effects of any of the variables of interest based on these weights. As a result, we have chosen to provide unweighted results for simplicity of interpretation.

The survey experiment began by measuring covariates including demographic information, political beliefs and affiliations, participants’ individual values and environmental attitudes. Participants were told that the California Department of Fish and Wildlife (CDFW) was considering moving forward with a proposal to manage and ultimately eradicate invasive wild pigs (*Sus scrofa*) and that CDFW would like to know more about the public’s opinions and support or opposition for such a project. Participants saw one of five randomly assigned press releases, which included four treatment messages that use a full factorial two (attribute frame: ecological vs. economic) by two (outcome frame: gain vs. loss) design. The ecological gain treatment highlights benefits to native California ecosystems and species that would result from implementation of the pig management program; the ecological loss treatment highlights further loss of native habitat and species destruction if CDFW fails to implement the management program; the economic gain treatment highlights the increase in statewide economic production and government tax revenue that would result from implementation of the management program; and the economic loss treatment highlights the continued loss of economic production and government tax revenue that would result from failure to implement the management program. The language from each fictional press release is provided in S1 Text. Treatment and Control Language Used. A fifth control condition was also included, which provided participants with information regarding CDFW’s planned implementation of the project but excluded project goals related to ecological or economic gains and losses. Although the press releases that were used in the experiment were fictional, they were modeled after real CDFW invasive species communications. Moreover, wild pigs do pose major economic and ecological problems for the state of California and plans to address the problem are being evaluated.

After reading the randomly assigned press release, participants were asked whether they supported or opposed the project and how strongly they held this position. Responses to these questions are the primary outcome measures. Participants were then told that CDFW was in the process of taking public comment on the project, and participants were asked to provide brief comments regarding why they supported or opposed the project. Whether they did so was used as a measure of political activism. Participants were provided debriefing information and the survey experiment was completed. Several attention checks were used throughout the survey experiment. Responses from any participant who spent less than 33% or more than 300% of mean survey response time were excluded from analysis. In addition, two separate attention check questions were used in which participants were asked to click a specific multiple choice option. Participants who failed either attention check questions were excluded. Additional questions related to a different project were also asked, but are not reported here [70].

### Measures

#### Support for invasive species management

The primary dependent variable used in the analysis is support for the wild pig management project described in each message frame. To increase the perceived importance and personal connection to the question, participants were asked “As a California resident, do you support or oppose the proposal…” Responses were measured as binary (support/oppose).

#### Strength of support for and opposition to invasive species management

A secondary dependent variable used in analysis is the strength of participants’ support for the project (if they were supportive) or opposition to the project (if they opposed it). These were measured by asking participants who supported the project were asked to indicate the level of strength of that support, with response options including “strongly support,” “support,” and “only slightly support.” Participants who opposed the project were similarly asked to indicate their strength of opposition, with options “strongly oppose,” “oppose,” and “only slightly oppose.” For analysis, we then coded these responses into a single ordinal measure of strength of support, from “strongly oppose” (1) to “strongly support” (6). Strength of an opinion or attitude, rather than just direction, helps explain the relationship between that attitude and behavior, which may include electoral decisions, participation in non-electoral political actions, or how opinion persistence [71–73].

#### Manipulation checks

We asked participants several questions to measure whether the treatments had influenced their thinking. First, we asked all participants whether wild pigs primarily present a problem to California because of their economic or ecological consequences. Participants could also select that they did not know. Results show a significant difference in response choice based on ecological or economic treatment frame (χ^2^(8) = 167.46, *p* < .01). Participants were then asked whether the program would “prevent further declines” or “allow for increases” in native species and habitat, for those who received an ecological treatment; or whether the program would “prevent further economic damages” or “allow additional economic benefits,” for those receiving an economic treatment. Responses were combined across the economic and ecological conditions and evaluated whether people were able to successfully identify the outcome frame they received. Results indicate that responses differed by gain or loss outcome frame (χ^2^(1) = 14.48, *p* < .01).

#### Heterogeneous treatment effects

Party identification and environmental values are used to test hypotheses regarding heterogeneous treatment effects. Environmental values were measured using an abridged version of the New Ecological Paradigm (NEP) [74,75]. This version of the NEP includes five questions, which were then combined into a single measure of environmental values (five items, Cronbach’s α = .64). In spite of only modest internal consistency in this study, and past research questioning the validity of the NEP as a unidimensional measure of environmental attitudes [76], use of the NEP provides a well-understood means of incorporating environmentalism into survey data. As a result, all responses were included in a single measure of NEP for analysis, including as a measure in subgroup analysis to determine whether environmentalists responded to treatments differently than non-environmentalists. For subgroup analyses, environmentalists were respondents with NEP scores in the top quartile, while non-environmentalists had responses in the bottom quartile.

Political ideology was included in models to account for possible influences of general political beliefs about government on support for a government program like invasive species management measured on a seven-point Likert scale, from “extremely liberal” (1) to “extremely conservative” (7). To evaluate subgroup effects by political ideology, we subset participants into liberals, conservatives, and moderates. Liberals are defined as anyone who responded they were “extremely liberal,” “liberal,” or “somewhat liberal” on the Likert scale (1–3). Conservatives are defined as participants who self-identified as “extremely conservative,” “conservative,” or “somewhat conservative” on the Likert scale (5–7). Moderates indicated they were “moderate; middle of the road” on the scale (4). Inclusion of these measures also allowed us to evaluate the extent to which variation in support for the wild pig management project overall was based on political affiliations or beliefs.

#### Controls

Several other variables were used as controls but are not the subject of detailed analysis. These included participants’ concern for animals’ well-being (four items, Cronbach’s α = .61); party identification, measured by asking participants whether they identify as a member of a particular party; education, measured by asking participants to identify their highest level of education achieved, from “Did not finish High School” (1) to advanced degrees (8); and annual household income, measured on an ordinal scale, from “Less than $20,000” to “Over $150,000.” The survey matched household income quotas that were consistent with existing U.S. Census information for California residents. Information on participants’ race/ethnicity, gender, age, and whether they live in a rural or urban environment were also measured and included in the models described below. All analysis was done in R, version 3.5.0 [77].

## Results

### Support for invasive species management

Support for invasive species management overall was high, with 73% of all respondents indicating that they would support the program outlined in the CDFW press release. We began analysis by estimating the effects of treatment assignment on support by specifying a logistic regression model. Predicted probabilities of support for the pig management program for each treatment condition and for changes across the two covariates significantly correlated with support are shown in Table 1. Table 1 and all other tables provided below control for covariates; they are not all shown. Full results, including for all covariates, can be found in S2 Fig.

**Table 1:** Effects of treatment on support for wild pig management.

Treatment/Covariate	Predicted Probability (pp)	Average Treatment Effect (ATE)^a^	N
Treatment—Control	0.68	-	218
Treatment—Ecological Gain	0.87	19pp^b^	215
Treatment—Ecological Loss	0.79	11pp^b^	217
Treatment—Economic Loss	0.76	8pp	216
Treatment—Economic Gain	0.73	5pp	211
Animal Welfare—Low Support	0.8	-	275
Animal Welfare—High Support	0.72	-8pp	269
Gender—Female	0.69	-	687
Gender—Male	0.83	14pp^b^	390

^a^ Results are predicted probabilities of support for the project. For manipulated variables, the ATE is the change in predicted probability (pp) for treatments in relation to control. In the logistic regression, animal welfare is included as a continuous variable and has a significant effect at *p <* .05. However, to show change in predicted probability based on animal welfare, we constructed high- and low-support measures with the top and bottom quartiles of respondents.

^b^ Effect is significant at *p <* .05 in logistic model.

Both ecological gain and ecological loss frames had positive and significant effects on support for invasive species management, with the ecological loss frames having the largest effect on overall support for the project. Neither of the economic frames had a significant effect on project support, though in both cases the direction of the effect was positive. Only two covariates–concern for animal welfare and gender–were significantly correlated with support for the project. People who were more concerned about animal welfare were less supportive of the project, likely because they find the prospect of killing animals, even invasive species, to be unacceptable. Men were more supportive of the project than women, which is consistent with previous invasive species opinion research [32,78].

Next, we pooled treatments into ecological or economic frames and gain or loss frames in order to evaluate the independent effects of each. A full model estimating pooled effects that includes control variables can be seen in S3 Fig. The pooled results shown in Table 2 show support for the invasive species management project differed significantly by attribute frame (χ^2^(4) = 29.22, *p* < .001). *H1* proposed that support for invasive species management would increase more when people are presented with an ecological message frame than with an economic frame. As anticipated, people who read the ecological message were more supportive of the invasive species management project than either people who read the control message (Kruskal-Wallis χ^2^(1) = 21.45, *p* < .001) or the economic message (K-W χ^2^(1) = 10.16, *p* = .004). When referencing comparisons of effects of different treatments to one another, we use a Bonferroni correction to *p <* .*05*. When *p-values* are reported, they are Bonferroni-adjusted. Support for the wild pig management program did not differ significantly between economic frames and the control frame (K-W χ^2^(1) = 3.66, *p* = .17).

**Table 2:** Effects of pooled treatments on support for wild pig management.

Treatment (Pooled)	Predicted Probability (pp)^a^	Average Treatment Effect (ATE)	N
Control	0.68	-	218
Ecological Treatments	0.83	15pp^b^	432
Economic Treatments	0.74	6pp	427
Loss Treatments	0.82	14pp^b^	426
Gain Treatments	0.76	8pp^b^	433

^a^ Results are predicted probabilities of support for the project in relation to control.

^b^ Significant at *p <* .05.

Outcome frames, which referenced benefits as either gains or avoided losses resulting from the program, also had significant effects on participants’ support of the project (χ^2^(2) = 19.52, *p* < .001). Support for the project was significantly greater among people who read the loss frames than the control frame (K-W χ^2^(1) = 19.21, *p* < .001). The logistic model suggested gain frames had a positive effect on project support as compared to the control message as well; however, when controlling for family-wise error the effect of gain frames does not remain significant (K-W χ^2^(1) = 4.64, *p* = .09). Loss frames were significantly more effective at increasing support than gain frames (K-W χ^2^(1) = 6.95, *p* < .05). Consistent with H6, loss frames are more effective than gain frames across the entire sample.

Together, these results indicate that people were more responsive to messages regarding the ecological benefits of managing invasive species than economic benefits. Results also indicate that people were more responsive to messages highlighting opportunities to prevent further losses than those highlighting comparable gains; consistent with these findings, the single most effective frame across the entire sample was the ecological loss frame.

Next, we evaluate whether treatments influenced the strength of support or opposition to the proposed invasive species management project. Understanding whether certain message frames increase how strongly people feel about an issue provides an important test of their potential influence on policy outcomes, in part because it informs how likely a person’s opinion on a particular issue is to influence their political behavior [71]. To evaluate the effects of different messages on strength of support, we specify an ordered logistic regression with the same predictors as the model used to produce predicted probabilities of support shown in Table 1. Odds ratios across the range of response options in each treatment condition are shown in Table 3 and indicate that the effect of different message frames on strength of support for the project reflect their effects on overall support.

**Table 3:** Effects of message frames on strength of support for wild pig management.

Message Frame	Odds Ratio	Standard Error	p-value
Ecological Loss	2.01^a^	0.2	< .001
Ecological Gain	1.51^a^	0.2	0.04
Economic Loss	1.18	0.2	0.42
Economic Gain	0.99	0.2	0.96

^a^ Significant at *p <* .05.

Ecological loss and ecological gain frames both had positive and significant effects on strength of support for the project described in the messages. The numbers in Table 3 can be interpreted as the increased likelihood of being one level higher on the ordered strength of support variable due to the treatment, as compared to the control group. People who read the ecological loss message were over twice as likely (odds ratio of 2.01) to have indicated "strongly support" than "support" (or to have indicated “support” rather than “neutral”), as compared to the control. Results regarding strength of support provide additional evidence that ecological messages are more effective than economic ones for changing opinion regarding invasive species management.

### Treatment-by-covariate heterogeneous effects

Next, we evaluate how different message frames influenced support for invasive species management among subgroups of participants, including among people with different political ideologies and people with different environmental values. Table 4 summarizes heterogeneous average treatment effects (ATEs) of pooled treatments based on respondents’ ideology. Ecological treatments had a positive and large effect on project support among political liberals and moderates, while economic treatments had no effect. When compared to economic messages, ecological messages significantly increased support for the project among political liberals (K-W χ^2^(1) = 7.29, *p* < .01) and moderates (K-W χ^2^(1) = 5.50, *p* < .05). This is consistent with expectations outlined in *H2*, that political liberals would be more responsive to ecological frames than economic frames. Among political conservatives, most treatments significantly increased likelihood of support the project as compared to those who read the control message, but there was no statistical difference in support for the project between conservatives receiving the ecological or economic messages (K-W χ^2^(1) = 0.7, *p* = .79). This is inconsistent with expectations outlined in *H3*; we anticipated conservatives would be likely more likely to support the project if they read an economic message than if they were exposed to the ecological message. Overall, our findings regarding the effects of gain and loss messages were mostly consistent with expectations that they would not significantly differ among different political groups.

**Table 4:** Average treatment effects by respondent ideology.

Ideology^a^	Treatment (Pooled)	Predicted Support^b^	Average Treatment Effect (ATE)	N
Liberals	Control	0.7	-	83
	Ecological Message	0.89	19pp^c^	164
	Economic Message	0.74	4pp	197
	Loss Message	0.83	13pp^c^	172
	Gain Message	0.79	9pp	189
Conservatives	Control	0.68	-	63
	Ecological Message	0.79	11pp	114
	Economic Message	0.83	15pp^c^	118
	Loss Message	0.81	13pp^c^	115
	Gain Message	0.8	12pp	117
Moderates	Control	0.68	-	56
	Ecological Message	0.82	14pp^c^	125
	Economic Message	0.74	6pp	93
	Loss Message	0.86	18pp^c^	117
	Gain Message	0.69	1pp	101

^a^ Ideology measured on a 7-point Likert scale. Moderates were defined as respondents who indicated they were “Moderate; Middle of the Road” (4). Liberals are defined as respondents who indicated they were “Extremely Liberal,” “Liberal,” or “Somewhat Liberal.” Conservatives are defined as respondents who indicated they were “Extremely Conservative,” “Conservative,” or “Somewhat Conservative.”

^b^ Results are predicted probabilities of support for the project in relation to control.

^c^ Significant at *p* < .05

There were no differences in support for invasive species management based on gain or loss frames among either conservatives (K-W χ^2^(1) = 0.48, *p* = .48) or moderates (K-W χ^2^(1) = 0.09, *p* = .76). However, liberals were significantly more supportive of invasive species management if faced with potential losses than when confronted with a message highlighting comparable gains (K-W χ^2^(1) = 5.57, *p* < .05). Heterogeneous treatment effects by party identification (as distinct from political ideology) are provided in S4 Fig, and show heterogeneous effects for political party is consistent with the effects by political ideology.

Table 5 shows average treatment effects among people with different environmental values. For environmentalists, ecological messages had significant positive effects on support for the project, while economic messages had no significant effects on support. This suggests that people who are most concerned with environmental protection are not only more responsive to messaging focused on benefits to nature, they are also indifferent toward economic messages. To test this relationship directly, we compared the effects of ecological messages directly to economic messages among environmentalists, which demonstrated that ecological messages were significantly more effective than economic ones among this group (K-W χ^2^(1) = 13.53, *p* < .001). This result is consistent with expectations outlined in *H4* that environmentalists will be more responsive to ecological frames.

**Table 5:** Average treatment effects by environmental values.

Environmentalism^a^	Treatment (Pooled)	Predicted Support^b^	Average Treatment Effect (ATE)	N
Environmentalists	Control	0.72	-	117
	Ecological Message	0.87	15pp^c^	221
	Economic Message	0.75	3pp	245
	Loss Message	0.83	11pp^c^	233
	Gain Message	0.78	6pp	233
Non-Environmentalists	Control	0.63	-	100
	Ecological Message	0.79	16pp^c^	211
	Economic Message	0.75	12pp	180
	Loss Message	0.81	18pp^c^	191
	Gain Message	0.74	11pp	200

^a^ Environmentalists are defined as respondents who scored above the median on the New Ecological Paradigm (NEP). Non-environmentalists are those respondents who scored below the median on the NEP.

^b^ Results are predicted probabilities of support for the project in relation to control.

^c^ Significant at *p* < .05

Among non-environmentalists, both ecological messages and economic messages significantly increased support for invasive species management, as compared to the control message. There was no significant difference in support among non-environmentalists based on whether they received the economic or ecological message (K-W χ^2^(1) = 0.62, *p* = .43), which contradicts expectations outlined in *H5* that economic messages would be more effective than ecological messages among non-environmentalists. We assumed that there would be some backlash against stated eco-centric goals among people who would not be expected to care about ecological outcomes and who might perceive ecological goals as threatening economic growth, but this does not appear to be the case.

As with all other subgroups, loss frames significantly increased support for invasive species management among both environmentalists and non-environmentalists as compared to control messages. However, when comparing gain and loss messages, results were more mixed. Loss messages were significantly more effective among non-environmentalists K-W χ^2^(1) = 3.85, *p* < .05), but did not have a significant effect on support among environmentalists K-W χ^2^(1) = 2.97, *p* = .09). The main hypotheses and the p-values associated with the appropriate Kruskal-Wallis test statistics are presented in Table 6.

**Table 6:** Main hypotheses and summary of findings.

	Main Hypotheses	Kruskal-Wallis Test Statistic
H1	Ecological more effective than economic frame	*p* = .004
H2	Liberals: Ecological more effective than economic frames	*p* < .01
H3	Conservatives: Economic more effective than ecological frames	*p* = .79
H4	Environmentalists: Ecological more effective than economic frames	*p* < .001
H5	Non-environmentalists: Economic more effective than ecological frames	*p* = .43
H6	Loss frames more effective than gain frames	*p* < .05

## Discussion

This study provides new evidence regarding how people think about and respond to environmental messages. The study provides support for loss aversion applied to public goods; participants were more supportive of invasive species management when that management was framed as preventing ecological or economic losses than if presented as offering equivalent potential ecological or economic gains. The greater impact of loss messages may also have been a result of the tendency for loss to increase issue salience compared to a focus on gains. We expected issue salience to be an important factor determining support for invasive species management due the public’s low overall awareness of invasive species issues. We also found that ecological-loss messages were more effective than economic-loss messages, which we hypothesize may result in part due to a sense that ecological impacts may feel more permanent, and thus both more salient and riskier, than economic losses feel. We suggest that the riskiness of impacts to native species is an important factor influencing people’s willingness to support new policy solutions for this issue [79], which contributes to ecological loss aversion.

The efficacy of ecological and economic frames in this study differed based on individuals’ political ideologies and environmentalism. Liberals were most responsive to ecological messaging, while conservatives were most responsive to economic frames. Somewhat surprisingly, we found that political moderates’ support for managing invasive species was increased by ecological messaging but not economic messaging. This result is in contrast to previous studies and prevailing wisdom that the ever-increasing polarization of environmental discourse means messages about must highlight the economic or other co-benefits of environmental policies rather than direct benefits to ecosystems [14,15]. The absence of a dominant political rhetoric regarding invasive species management may have contributed to this result. For other environmental issues–climate change, habitat conservation, oil and gas drilling–people tend to rely on their existing partisan identities to shape their opinions; the absence of this partisan divide for invasive species provides more space for a range of justifications to support environmental management, including ecological benefit.

It is surprising that ecological messages were equally effective among environmentalists and non-environmentalists. While support for invasive species management among environmentalists was significantly influenced by ecological messages but unchanged by economic messages, both ecological and economic messages increased support among non-environmentalists. This indicates even more strongly that economic arguments to support environmental management may not be essential for all issues, including among people whose politics or values do not otherwise align with typical environmental priorities.

This study has several limitations. First, the results presented here are likely shaped by low awareness about the impacts and extent of invasive species [80]. We did not ask respondents for their knowledge about invasive species or wild pigs and their associated impacts, and therefore did not control for prior knowledge or test whether there were heterogeneous treatment effects of messaging by prior knowledge. Such an analysis could be useful in the future. Second, the treatment messages in this study included a proposed lethal eradication program. Because public support for invasive species management has been found to be significantly higher for non-lethal programs [37], it is likely that support for management would be even higher for non-lethal proposals. Third, the study was conducted on California residents, who may differ in important ways from residents of other states. Each of these limitations suggests caution is warranted when generalizing the results to other species, other programs, and other populations.

This paper offers important considerations for environmental policy advocates and managers considering how to effectively message environmental priorities. While we focused on only a single issue of environmental management, our findings advance overall understanding of how environmental communications influence environmental public opinion. Most research to date on the subject has focused on climate change communications, which are important but may not provide transferable lessons for many environmental issues. Invasive species management shares characteristics of issues such as wildlife and ecosystem conservation, land use, valuation of ecosystem services, and many others in which humans manage the environment for both our own and ecological impacts.

## Supporting information

S1 Fig Sample demographics. (PDF)

S2 Fig Complete models comparing binary support and strength of support for invasive species management. (PDF)

S3 Fig Models estimating pooled treatment effects on support for invasive species management. (PDF)

S4 Fig Models estimating heterogeneous treatment effects by party identification. (PDF)

S1 Text Treatment and control language used. (PDF)

S1 Data Dataset from survey experiment. (CSV)

## References

[1] De Groot R, Brander L, Van Der Ploeg S, Costanza R, Bernard F, Braat L, et al Global estimates of the value of ecosystems and their services in monetary units. Ecosyst Serv. 2012;1(1):50–61.

[2] Costanza R, d’Arge R, De Groot R, Farber S, Grasso M, Hannon B, et al The value of the world’s ecosystem services and natural capital. Nature. 1997;387(6630):253.

[3] Costanza R, De Groot R, Farber S, Grasso M, Hannon B, Limburg K, et al The value of the world# s ecosystem services and natural capital. Ecol Econ. 1998;25(1):3–15.

[4] Daniels DP, Krosnick JA, Tichy MP, Tompson T. Public opinion on environmental policy in the United States. Handb US Environ policy. 2012;461–86.

[5] Guber DL. The Grassroots of a Green Revolution: Polling American on the Environment. Cambridge, MA: MIT Press; 2002.

[6] Speth JG. Foreward In: Repetto R, editor. Punctuated equilibrium and the dynamics of US environmental policy. New Haven, CT: Yale University Press; 2006.

[7] Page BI, Shapiro RY. The rational public: Fifty years of trends in Americans’ policy preferences. Chicago, IL: University of Chicago Press; 1992.

[8] Bertolino S, Genovesi P. Spread and attempted eradication of the grey squirrel (Sciurus carolinensis) in Italy, and consequinces for the red squirrel (Sciurus vulgaris) in Eurasia. Biol Conserv. 2002;109:351–8.

[9] Beierle TC. Using Social Goals to Evaluate Public Participation in Environmental Decisions. Policy Stud Rev. 1999;16(3–4):75–103.

[10] Eden S. Public participation in environmental policy: considering scientific, counter-scientific and non-scientific contributions. Public Understand Sci. 1996;5:183–204.

[11] O’Faircheallaigh C. Public participation and environmental impact assessment: Purposes, implications, and lessons for public policy making. Environ Impact Assess Rev. 2010;30(1):19–27.

[12] Bolsen T. The construction of news: Energy crises, advocacy messages, and frames toward conservation. Int J Press. 2011;16(2):143–62.

[13] Ansolabehere S, Konisky DM. The American public’s energy choice. Daedalus. 2012;141(2):61–71.

[14] Spence A, Pidgeon N. Framing and communicating climate change: The effects of distance and outcome frame manipulations. Glob Environ Chang. 2010;20(4):656–67.

[15] Bertolotti M, Catellani P. Effects of message framing in policy communication on climate change. Eur J Soc Psychol. 2014;44(5):474–86.

[16] Tanentzap AJ, Kirby KJ, Goldberg E. Slow responses of ecosystems to reductions in deer (Cervidae) populations and strategies for achieving recovery. For Ecol Manage. 2012;264:159–66.

[17] Crowley SL, Hinchliffe S, McDonald RA. Conflict in invasive species management. Front Ecol Environ. 2017 4;15(3):133–41.

[18] McNeely JA. The Great Reshuffling: Human dimensions of alien invasive species. 2001 1–235 p.

[19] Popkin SL. The Reasoning Voter: Communication and Persuasion in Presidential Campaigns. 2nd ed Chicago, IL: University of Chicago Press; 1994.

[20] Nelson TE, Oxley ZM, Clawson RA. Toward a psycology of framing effects. Polit Behav. 1997;19(3):221–46.

[21] Nelson TE, Oxley ZM. Issue Framing Effects on Belief Importance and Opinion. J Polit. 1999;61(4):1040–67.

[22] Nisbet MC, Markowitz EM, Kotcher JE. Winning the Conversation: Framing and Moral Messaging in Environmental Campaigns. Talk Green Explor Curr issues Environ Commun. 2012;9–36.

[23] Kahneman D, Tversky A. Prospect Theory: An Analysis of Decision under Risk. Econometrica. 1979;47(2):263–92.

[24] Kahneman D, Tversky A. Choices, Values, and Frames. Am Psychol. 1984;39(4):341–50.

[25] Levin IP, Schneider SL, Gaeth GJ. All Frames Are Not Created Equal: A Typology and Critical Analysis of Framing Effects. Organ Behav Hum Decis Process. 1998;76(2):149–88. 9831520 10.1006/obhd.1998.2804

[26] Bradshaw CJA, Leroy B, Bellard C, Roiz D, Albert C, Fournier A, et al Massive yet grossly underestimated global costs of invasive insects. Nat Commun. 2016 10;7(September 16):12986 10.1038/ncomms12986 27698460 PMC5059451

[27] Sala OE, Chapin FSI, Armesto JJ, Berlow E, Bloomfield J, Dirzo R, et al Global Biodiversity Scenarios for the Year 2100. Science (80-). 2000;287(5459):1770–5. 10.1126/science.287.5459.1770 10710299

[28] Pimentel D, Zuniga R, Morrison D. Update on the environmental and economic costs associated with alien-invasive species in the United States. Ecol Econ. 2005;52(3 SPEC. ISS.):273–88.

[29] Gurevitch J, Padilla D. Are invasive species a major cause of extinctions? Trends Ecol Evol. 2004;19(9):470–4. 10.1016/j.tree.2004.07.005 16701309

[30] Doherty TS, Glen AS, Nimmo DG, Ritchie EG, Dickman CR. Invasive predators and global biodiversity loss. Proc Natl Acad Sci. 2016;113(40):201602480. 10.1073/pnas.1602480113 PMC5056110 27638204

[31] Sharp RL, Larson LR, Green GT. Factors influencing public preferences for invasive alien species management. Biol Conserv. 2011 8;144(8):2097–104.

[32] Bremner A, Park K. Public attitudes to the management of invasive non-native species in Scotland. Biol Conserv. 2007 10;139(3–4):306–14.

[33] Simberloff D., Parker IM., Windle PN. Introduced species policy, management, and future research needs. Front Ecol Environ. 2005;3(1 SPEC. ISS.):12–20.

[34] Moser KW, Barnard EL, Billings RF, Crocker SJ, Dix ME, Gray AN, et al Impacts of Nonnative Invasive Species on US Forests and Recommendations for Policy and Management. J For. 2009;(September):320–7.

[35] Dunlap RE, Xiao C, McCright AM. Politics and environment in America: Partisan and ideological cleavages in public support for environmentalism. Env Polit. 2001;10(4):23–48.

[36] Feygina I, Jost JT, Goldsmith RE. System justification, the denial of global warming, and the possibility of “system-sanctioned change.” Personal Soc Psychol Bull. 2010;36(3):326–38. 10.1177/0146167209351435 20008965

[37] Selge S, Fischer A, van der Wal R. Public and professional views on invasive non-native species–A qualitative social scientific investigation. Biol Conserv. 2011 12;144(12):3089–97.

[38] Veitch C, Clout M. Turning the tide: the eradication of invasive species. Proc Int Conf Erad Isl Invasives. 2002;(27):155–64.

[39] Marshall GR, Coleman MJ, Sindel BM, Reeve IJ, Berney PJ. Collective action in invasive species control, and prospects for community-based governance: The case of serrated tussock (Nassella trichotoma) in New South Wales, Australia. Land use policy. 2016;56.

[40] Cantrill JG. Communication and Our Environment: Categorizing Research in Environmental Advocacy. Vol. 21, Journal of Applied Communication Research. 1993 66–95 p.

[41] Davis JJ. The effects of message framing on response to environmental communications. Journal Mass Commun Q. 1995;72:285–99.

[42] Lakoff G. Why it Matters How We Frame the Environment. Environ Commun. 2010;4(1):70–81.

[43] Gallup. Most Important Problem [Internet]. 2019. Available from: https://news.gallup.com/poll/1675/most-important-problem.aspx

[44] Peterson TD, Rose AZ. Reducing conflicts between climate policy and energy policy in the US: The important role of the states. Energy Policy. 2006;

[45] Selin H, VanDeveer SD. Political science and prediction: What’s next for U.S. climate change policy? Rev Policy Res. 2007;

[46] Vezirgiannidou S-E. Climate and energy policy in the United States: the battle of ideas. Env Polit. 2013;22(4):593–609.

[47] Sacchi S, Riva P, Brambilla M, Grasso M. Moral reasoning and climate change mitigation: The deontological reaction toward the market-based approach. J Environ Psychol. 2014;

[48] Markowitz EM, Shariff AF. Climate change and moral judgement. Nat Clim Chang. 2012;2(4):243–7.

[49] Graham J, Haidt J, Nosek BA. Liberals and Conservatives Rely on Different Sets of Moral Foundations. J Pers Soc Psychol. 2009;96(5):1029–46. 10.1037/a0015141 19379034

[50] Schwartz SH, Caprara GV, Vecchione M. Basic Personal Values, Core Political Values, and Voting: A Longitudinal Analysis. Polit Psychol. 2010;31(3):421–52.

[51] Feinberg M, Willer R. The Moral Roots of Environmental Attitudes. Psychol Sci. 2013;24(1):56–62. 10.1177/0956797612449177 23228937

[52] McCright AM, Dunlap RE. Cool dudes: The denial of climate change among conservative white males in the United States. Glob Environ Chang. 2011;21(4):1163–72.

[53] Clifford S, Jerit J. How words do the work of politics: Moral foundations theory and the debate over stem cell research. J Polit. 2013;75(3):659–71.

[54] Saatchi S, Mascaro J, Xu L, Keller M, Yang Y, Duffy P, et al Seeing the forest beyond the trees. Vol. 24, Global Ecology and Biogeography. 2015 p. 606–10.

[55] Gonzales MH, Aronson E, Costanzo MA. Using Social Cognition and Persuasion to Promote Energy Conservation: A Quasi‐Experiment. J Appl Soc Psychol. 1988;18(12):1049–66.

[56] Novemsky N, Kahneman D. The Boundaries of Loss Aversion. J Mark Res. 2005;42(2):119–28.

[57] Cobb MD. Framing effects on public opinion about nanotechnology. Sci Commun. 2005;27(2):221–39.

[58] Loroz PS. The Interaction of Message Frames and Reference Points in Prosocial Persuasive Appeals. Psychol Mark. 2007;24(11):1001–23.

[59] Hardisty DJ, Weber EU. Discounting future green: Money versus the environment. J Exp Psychol Gen. 2009;138(3):329–40. 10.1037/a0016433 19653793

[60] White K, MacDonnell R, Dahl DW. It’s the Mind-Set That Matters: The Role of Construal Level and Message Framing in Influencing Consumer Efficacy and Conservation Behaviors. J Mark Res. 2011;48(3):472–85.

[61] Cheng T, Woon DK, Lynes JK. The Use of Message Framing in the Promotion of Environmentally Sustainable Behaviors. Soc Mar Q. 2011;17(2):48–62.

[62] Meyerowitz BE, Chaiken S. The effect of message framing on breast self-examination attitudes, intentions, and behavior. J Pers Soc Psychol. 1987;52(3):500–10. 3572721 10.1037//0022-3514.52.3.500

[63] Quattrone GA, Tversky A. Causal versus diagnostic contingencies: On self-deception and on the voter’s illusion. J Pers Soc Psychol. 1984;46(2):237–48.

[64] Weitzman ML. On modeling and interpreting the economics of catastrophic climate change. Rev Econ Stat. 2009;91(1):1–19.

[65] Costello CJ, Neubert MG, Polasky SA, Solow AR. Bounded uncertainty and climate change economics. Proc Natl Acad Sci. 2010;107(18):8108–10. 10.1073/pnas.0911488107 20404189 PMC2889512

[66] Keller RP, Frang K, Lodge DM. Preventing the spread of invasive species: Economic benefits of intervention guided by ecological predictions. Conserv Biol. 2008;22(1):80–8. 10.1111/j.1523-1739.2007.00811.x 18254855

[67] Keller RP, Lodge DM, Lewis MA, Shogren JF, editors. Bioeconomics of Invasive Species: Integrating ecology, economics, policy, and management. Oxford, UK: Oxford University Press; 2009.

[68] Pejchar L, Mooney HA. Invasive species, ecosystem services and human well-being. Trends Ecol Evol. 2009;24(9):497–504. 10.1016/j.tree.2009.03.016 19577817

[69] Bain PG, Hornsey MJ, Bongiorno R, Jeffries C. Promoting pro-environmental action in climate change deniers. Nat Clim Chang. 2012;2(8):600–3.

[70] Hiroyasu EHT, Miljanich CP, Anderson SE. Drivers of support: The case of species reintroductions with an ill-informed public. Hum Dimens Wildl [Internet]. 2019 5 29;1–17. Available from: https://www.tandfonline.com/doi/full/10.1080/10871209.2019.1622055

[71] Krosnick JA, Abelson RP. The case for measuring attitude strength in surveys In: Questions about Questions: Inquiries into the cognitive basis of surveys. New York, NY: Russell Sage Foundation; 1992 p. 177–203.

[72] Fazio RH, Zanna MP. Attitudinal qualities relating to the strength of the attitude-behavior relationship. J Exp Soc Psychol. 1978;14(4):398–408.

[73] Fazio RH, Zanna MP. On the predictive validity of attitudes: The roles of direct experience and confidence. J Pers. 1978;46(2):228–43.

[74] Dunlap RE, Van Liere KD, Mertig AG, Jones RE. New Trends in Measuring Environmental Attitudes: Measuring Endorsement of the New Ecological Paradigm: A Revised NEP Scale. J Soc Issues. 2000;56(3):425–42.

[75] Stern PC, Dietz T, Abel T, Guagnano GA, Kalof L. A value-belief-norm theory of support for social movements: The case of environmentalism. Hum Ecol Rev. 1999;6(2):81–97.

[76] Amburgey JW, Thoman DB. Dimensionality of the New Ecological Paradigm: Issues of factor structure and measurement. Environ Behav. 2012;44(2):235–56.

[77] R Development Core Team. R: A Language and Environment for Statistical Computing Team RDC, editor. R Foundation for Statistical Computing. Vienna, Austria: R Foundation for Statistical Computing; 2018 p. 409 (R Foundation for Statistical Computing; vol. 1).

[78] Fitzgerald G, Fitzgerald N, Davidson C. Public attitudes towards invasive animals and their impacts. 2007.

[79] Schüttler E, Rozzi R, Jax K. Towards a societal discourse on invasive species management: A case study of public perceptions of mink and beavers in Cape Horn. J Nat Conserv. 2011 7;19(3):175–84.

[80] Wilson C, Tisdell C. What Role Does Knowledge of Wildlife Play in Providing Support for Species’ Conservation? J Soc Sci. 2005 1;1(1):47–51.

