# Supplementary material for: Economic losses or environmental gains? Framing effects on public support for environmental management
Source: PLoS One. 2019 Jul 25;14(7):e0220320. doi: 10.1371/journal.pone.0220320 (PMC6657883; doi:10.1371/journal.pone.0220320)
Supplement: S1 Fig — (PDF) [file pone.0220320.s001.pdf]

S1 Fig. Sample Demographics

Statistic	Sample Statistics	CA Pop. Statistics
Age		
18-25 yrs	16.5%	14.2%
26-35 yrs	23.8%	19.3%
36-50 yrs	22.0%	28.7%
51-65 yrs	25.1%	23.2%
>65 yrs	16.1%	14.6%
Gender		
Male	36.2%	49.7%
Female	63.8%	50.3%
Household Income		
<$40,000	35.7%	33.7%
$40,000-75,000	25.1%	20.8%
$75,000-150,000	24.9%	27.3%
>$150,000	14.4%	14.0%
Education		
HS Grad or Less	13.8%	38.6%
Some College	40.6%	29.1%
Bachelor's Degree	30.6%	20.3%
Advanced Degree	15.1%	12%
Total Bachelor's or Higher	45.7%	32.3%
Race		
Non-Hispanic White	56.2%	37.8%
Hispanic	20.1%	38.8%
Asian	17.3%	14%
Black	5.9%	5.6%
American Indian	3.7%	<1%
Household Location		
Rural	24.1%	13%
Urban/Suburban	75.9%	87%
Party ID		
Democrat	47.8%	44.8%
Republican	28%	27.3%
Independent	24.1%	23.3%

Data comes from 2010 and 2015 American Community Survey estimates, and from the Public Policy Institute of California Statewide Survey. CA population data for age are estimates, as age bins do not align between the ACS and our survey.
