# Supplementary material for: Economic losses or environmental gains? Framing effects on public support for environmental management
Source: PLoS One. 2019 Jul 25;14(7):e0220320. doi: 10.1371/journal.pone.0220320 (PMC6657883; doi:10.1371/journal.pone.0220320)
Supplement: S2 Fig — (PDF) [file pone.0220320.s002.pdf]

S2 Fig. Complete Models Comparing Binary Support and Strength of Support for Invasive Species Management.

	Binary Support for the Project (Logistic Regression)	Strength of Support for the Project (Ordered Logistic Regression)
Treatments		
Ecological Gain	0.57^{**}	0.54^{***}
	(0.23)	(0.18)
Ecological Loss	1.11^{***}	0.99^{***}
	(0.25)	(0.18)
Economic Gain	0.23	0.24
	(0.22)	(0.18)
Economic Loss	0.45^{**}	0.30
	(0.23)	(0.18)
Attitudes/Values		
NEP	0.12	0.21^{***}
	(0.10)	(0.07)
Animal Welfare	-0.36^{***}	-0.23^{***}
	(0.11)	(0.08)
Political Ideology	−0.04	−0.01
	(0.06)	(0.05)
Party ID: Democrat	−0.16	−0.22
	(0.20)	(0.15)
Party ID: Republican	−0.03	0.11
	(0.24)	(0.18)
Resources		
Education	−0.00	−0.02
	(0.06)	(0.04)
Household Income	−0.01	0.02
	(0.04)	(0.03)
Demographic Controls		
Gender: Female	-0.72^{***}	-0.70^{***}
	(0.17)	(0.13)
Rural	−0.05	0.07
	(0.19)	(0.15)
Race: Black	−0.24	−0.07
	(0.39)	(0.31)
Race: Latinx	−0.02	−0.02
	(0.21)	(0.16)
Race: Asian	0.36	0.11
	(0.22)	(0.16)
Race: Other	−0.12	−0.19
	(0.34)	(0.27)
Constant	2.84^{***}	
	(0.68)	
Observations	992	992
Log Likelihood	−537.92	
Akaike Inf. Crit.	1,111.85	3,124.41

Note: $^{*}p<0.1$; $^{**}p<0.05$; $^{***}p<0.01$
