# Supplementary material for: Economic losses or environmental gains? Framing effects on public support for environmental management
Source: PLoS One. 2019 Jul 25;14(7):e0220320. doi: 10.1371/journal.pone.0220320 (PMC6657883; doi:10.1371/journal.pone.0220320)
Supplement: S3 Fig — (PDF) [file pone.0220320.s003.pdf]

S3 Fig. Models Estimating Pooled Treatment Effects on Support for Invasive Species Management.

	Support for Invasive Species Management Project	
	(Logit)	(Logit)
Ecological Message	0.81***	
	(0.20)	
Economic Message	0.33*	
	(0.19)	
Gain Message		0.39**
		(0.19)
Loss Message		0.75***
		(0.20)
Attitudes		
NEP	0.13	0.11
	(0.10)	(0.10)
Animal Welfare	−0.38***	−0.37***
	(0.11)	(0.10)
Political Ideology	−0.04	−0.04
	(0.06)	(0.06)
Democrat	−0.16	−0.18
	(0.20)	(0.20)
Republican	−0.03	−0.04
	(0.24)	(0.24)
Resources		
Education	0.001	−0.001
	(0.06)	(0.06)
Income	−0.004	−0.01
	(0.04)	(0.04)
Demographic Controls		
Gender	−0.72***	−0.71***
	(0.17)	(0.17)
Rural	−0.05	−0.04
	(0.19)	(0.19)
Race; Black	−0.27	−0.24
	(0.39)	(0.38)
Race: Latinx	−0.03	−0.02
	(0.21)	(0.21)
Race:Asian	0.37*	0.38*
	(0.22)	(0.22)
Race: Other	−0.11	−0.08
	(0.33)	(0.33)
Constant	2.86***	2.91***
	(0.68)	(0.68)
Observations	992	992
Log Likelihood	−540.51	−542.13
Akaike Inf. Crit.	1,113.02	1,116.27
Note:		*p<0.1; **p<0.05; ***p<0.01
