# Supplementary material for: Economic losses or environmental gains? Framing effects on public support for environmental management
Source: PLoS One. 2019 Jul 25;14(7):e0220320. doi: 10.1371/journal.pone.0220320 (PMC6657883; doi:10.1371/journal.pone.0220320)
Supplement: S4 Fig — (PDF) [file pone.0220320.s004.pdf]

S4 Fig. Models Estimating Heterogeneous Treatment Effects By Political Ideology.

Treatment (Pooled)	Predicted Support	ATE	N
Liberals			
Control	.70	-	83
Ecological Message	.89	19pp*	164
Economic Message	.74	4pp	197
Loss Message	.83	13pp*	172
Gain Treatments	.79	9pp	189
Conservatives			
Control	.68	-	63
Ecological Message	.79	11pp	114
Economic Message	.83	15pp*	118
Loss Message	.81	13pp*	115
Gain Message	.80	12pp	117
Moderates			
Control	.68	-	56
Ecological Message	.82	14pp*	125
Economic Message	.74	6pp	93
Loss Message	.86	18pp*	117
Gain Message	.69	1pp	101

Notes: Ideology was measured on a 7-point Likert scale. Moderates were defined as people who responded "Moderate, Middle of the Road (4); Liberals were defined as those who responded "Extremely Liberal," "Liberal," or "Somewhat Liberal" (1-3); Conservatives were responded "Extremely Conservative," "Conservative," or "Somewhat Conservative" (5-7).
* significant at $p<.05$
