# Supplementary material for: Economic losses or environmental gains? Framing effects on public support for environmental management
Source: PLoS One. 2019 Jul 25;14(7):e0220320. doi: 10.1371/journal.pone.0220320 (PMC6657883; doi:10.1371/journal.pone.0220320)
Supplement: S1 Text — (PDF) [file pone.0220320.s005.pdf]

S5 Text. Treatment and Control Language Used

<u>Ecological Gain Message:</u>

Program to Eliminate Invasive Wild Pigs in California
For Immediate Release

February 8, 2017

California Department of Fish and Wildlife

Wild pigs or feral pigs (Sus scrofa) were first brought to California by Spanish settlers in the 1700s. Since then, and with many more introduction events, wild pigs have colonized almost every county in the state. Wild pigs are habitat generalists and occupy a variety of habitats, including native California grasslands, oak woodlands, and along creeks and streams.

Control measures have typically been limited to hunting pigs on both public and private lands, requiring the purchase of one tag per animal hunted. The California Department of Fish and Wildlife (CDFW) does not have an active management plan for invasive wild pigs. However, populations have become more and more established across the state, making control through hunting difficult. As of 2017, wild pigs present a major threat to native habitats and the food supply and survival of native California species.

As a result, CDFW is proposing a more rigorous approach to addressing the problem of invasive pigs. The proposal involves a program to trap and cull wild pig populations in every county in the state, with the ultimate goal of eliminating the entire wild pig population across the state.

The Department has identified **major ecological benefits associated with implementation of the program**:

- Increased populations of important native and endangered California species such as coastal elk that still exist and the native plants and reptiles that are eaten by wild pigs.
- Increased oak survival, aiding in efforts to preserve iconic habitat that is home to many native species.
- In total, successful implementation of the project will provide major benefits for nearly three dozen native California species that rely on the same food sources and live in the same habitats as the wild pigs.

CDFW is asking Californians' for their support and input on the project before planned early-stage implementation in Spring 2017.

Ecological Loss Message:

Press Release: Program to Eliminate Invasive Wild Pigs in California
For Immediate Release

February 8, 2017

California Department of Fish and Wildlife

Wild pigs or feral pigs (Sus scrofa) were first brought to California by Spanish settlers in the 1700s. Since then, and with many more introduction events, wild pigs have colonized almost every county in the state. Wild pigs are habitat generalists and occupy a variety of habitats, including native California grasslands, oak woodlands, and along creeks and streams.

Control measures have typically been limited to hunting pigs on both public and private lands, requiring the purchase of one tag per animal hunted. The California Department of Fish and Wildlife (CDFW) does not have an active management plan for invasive wild pigs. However, populations have become more and more established across the state, making control through hunting difficult. As of 2016, wild pigs present a major threat to native habitats and the food supply and survival of native California species.

As a result, CDFW is proposing a more rigorous approach to addressing the problem of invasive pigs. The proposal involves a program to trap and cull wild pig populations in every county in the state, with the ultimate goal of eliminating the entire wild pig population across the state.

The Department has identified **major ecological losses if the program is not implemented**:

- Continued decline in population numbers of important native and endangered California species such as coastal elk that still exist and the native plants and reptiles that are eaten by wild pigs.
- Increased oak death, leading to continued destruction of iconic habitat that is home to many native species.
- In total, the failure to implement the program could lead to further decline of three dozen native California species that rely on the same food sources and live in the same habitats as the wild pigs.

CDFW is asking Californians' for their support and input on the project before planned early-stage implementation in Spring 2017.

Economic Gain Message:

Press Release: Program to Eliminate Invasive Wild Pigs in California
For Immediate Release

February 8, 2017

California Department of Fish and Wildlife

Wild pigs or feral pigs (Sus scrofa) were first brought to California by Spanish settlers in the 1700s. Since then, and with many more introduction events, wild pigs have colonized almost every county in the state. Wild pigs are habitat generalists and occupy a variety of habitats, including native California grasslands, oak woodlands, and along creeks and streams.

Control measures have typically been limited to hunting pigs on both public and private lands, requiring the purchase of one tag per animal hunted. The California Department of Fish and Wildlife (CDFW) does not have an active management plan for invasive wild pigs. However, populations have become more and more established across the state, making control through hunting difficult. As of 2016, wild pigs present a major financial burden to farmers and ranchers across the state.

As a result, CDFW is proposing a more rigorous approach to addressing the problem of invasive pigs. The proposal involves a program to trap and cull wild pig populations in every county in the state, with the ultimate goal of eliminating the entire wild pig population across the state.

The Department has identified **major economic benefits associated with implementation of the program**:

- $1.5 billion annual increase in the value of agricultural sales statewide by eliminating the major damages to private farmland.
- Over $12 million increase in annual state revenue from the sale of hunting tags for other animals like deer and elk by reducing competition with pigs.
- In total, successful implementation of the project will mean $15 billion increased state GDP and $120 million increase in state revenue over the next ten years.

CDFW is asking Californians' for their support and input on the project before planned early-stage implementation in Spring 2017.

Economic Loss Message:

Press Release: Program to Eliminate Invasive Wild Pigs in California
For Immediate Release

February 8, 2017

California Department of Fish and Wildlife

Wild pigs or feral pigs (Sus scrofa) were first brought to California by Spanish settlers in the 1700s. Since then, and with many more introduction events, wild pigs have colonized almost every county in the state. Wild pigs are habitat generalists and occupy a variety of habitats, including native California grasslands, oak woodlands, and along creeks and streams.

Control measures have typically been limited to hunting pigs on both public and private lands, requiring the purchase of one tag per animal hunted. The California Department of Fish and Wildlife (CDFW) does not have an active management plan for invasive wild pigs. However, populations have become more and more established across the state, making control through hunting difficult. As of 2017, wild pigs present a major financial burden to farmers and ranchers across the state.

As a result, CDFW is proposing a more rigorous approach to addressing the problem of invasive pigs. The proposal involves a program to trap and cull wild pig populations in every county in the state, with the ultimate goal of eliminating the entire wild pig population across the state.

The Department has identified **major economic losses if the program is not implemented**:

- $1.5 billion annual loss in value of agricultural sales statewide due to continued damage to farm and rangeland.
- $12 million annual state revenue lost from potential sale of hunting tags for other animals like deer and elk that are out-competed by pigs.
- In total, failure to implement the project will mean $15 billion lost state GDP and $120 million lost state revenue over the next ten years.

CDFW is asking Californians' for their support and input on the project before planned early-stage implementation in Spring 2017.

Control Message:

Press Release: Program to Eliminate Invasive Wild Pigs in California
For Immediate Release

February 8, 2017

California Department of Fish and Wildlife

Wild pigs or feral pigs (Sus scrofa) were first brought to California by Spanish settlers in the 1700s. Since then, and with many more introduction events, wild pigs have colonized almost every county in the state. Wild pigs are habitat generalists and occupy a variety of habitats, including native California grasslands, oak woodlands, and along creeks and streams.

Control measures have typically been limited to hunting pigs on both public and private lands, requiring the purchase of one tag per animal hunted. The California Department of Fish and Wildlife (CDFW) does not currently have a management plan for invasive wild pigs. However, populations have become more and more established across the state, making control through hunting difficult. As of 2017, wild pigs present a major financial burden to farmers and ranchers across the state.

As a result, CDFW is proposing a more rigorous approach to addressing the problem of invasive pigs. The proposal involves a program to trap and cull wild pig populations in every county in the state, with the ultimate goal of eliminating the entire wild pig population across the state.

CDFW is asking Californians' for their support and input on the project before planned early-stage implementation in Spring 2017.
